# Expression of P-REX2a is associated with poor prognosis in endometrial malignancies

**DOI:** 10.18632/oncotarget.25349

**Published:** 2018-05-15

**Authors:** Sho Takeshita, Yoriko Yamashita, Kosuke Shiomi, Nako Suzuki, Jun Yoshida, Aya Naiki-Ito, Shugo Suzuki, Shinya Akatsuka, Shinya Toyokuni, Takashi Takahashi, Shoko Mase, Atsushi Arakawa, Mayumi Sugiura-Ogasawara, Satoru Takahashi

**Affiliations:** ^1^ Department of Obstetrics and Gynecology, Nagoya City University Graduate School of Medicical Sciences, Mizuho-cho, Mizuho-ku, Nagoya, 467-8601, Japan; ^2^ Department of Experimental Pathology and Tumor Biology, Nagoya City University Graduate School of Medical Sciences, Mizuho-cho, Mizuho-ku, Nagoya, 467-8601, Japan; ^3^ Department of Pathology and Biological Responses, Nagoya University Graduate School of Medicine, Showa-ku, Nagoya, 466-8550, Japan; ^4^ Division of Molecular Carcinogenesis, Center for Neurological Diseases and Cancer, Nagoya University Graduate School of Medicine, Showa-ku, Nagoya, 466-8550, Japan

**Keywords:** endometrial neoplasms, GTP/GDP exchange factors, immunohistochemistry, prognosis, PTEN

## Abstract

P-REX2a is a PTEN inhibitor that also activates Rac 1. No associations with P-REX2a and human endometrial cancers have been reported to date. In this study, we immunohistochemically analyzed 155 uterine endometrial malignancies for P-REX2a expression. The P-REX2a-positive tumors displayed worse prognosis independent of PTEN expression. Then, we transduced either P-REX2a expression vector or short hairpin RNAs targeting P-REX2a into 2 uterine endometrioid carcinoma cell lines, OMC-2 and JHUEM-14. Ectopic expression of P-REX2a led to increased cell proliferation only in the PTEN-expressing OMC-2 cells but did not show any change in the PTEN-negative JHUEM-14 cells or the P-REX2a-knockdown cells. Induction of P-REX2a increased and knockdown of P-REX2a decreased cell migration in both cell lines. Then, we performed expression microarray analysis using these cells, and pathway analysis revealed that the expression of members of the GPCR downstream pathway displayed the most significant changes induced by the knockdown of P-REX2a. Immunohistochemical analysis revealed that Vav1, a member of the GPCR downstream pathway, was expressed in 139 of the 155 endometrial tumors, and the expression levels of Vav1 and P-REX2a showed a positive correlation (*r* = 0.44, *p* < 0.001). In conclusion, P-REX2a enhanced cell motility via the GPCR downstream pathway independently of PTEN leading to progression of uterine endometrioid malignancies and poor prognosis of the patients.

## INTRODUCTION

The incidence of endometrial carcinoma has been increasing in recent years, and endometrial carcinoma accounts for 4.8% of cancer in women in the Western countries, which makes it the fifth most common type of cancer [1]. The known prognostic factors of endometrial carcinoma are mostly classical, including histological type and grade, invasion into the myometrium or lymphovascular spaces, and several immunohistochemical markers such as p53, Ki67, and oestrogen receptor, as well as DNA ploidy [2]. However, newly recognized molecules have been recently proposed as prognostic factors, such as the loss of expression of androgen receptor expression, receptor activator of nuclear factor kappa-B or L1-cell adhesion molecule, which can contribute to worse prognosis; alternatively, patients with mutations in POLE exonuclease domain or loss of PTEN expression have been shown to have better outcomes [3–6].

**Figure 1:**
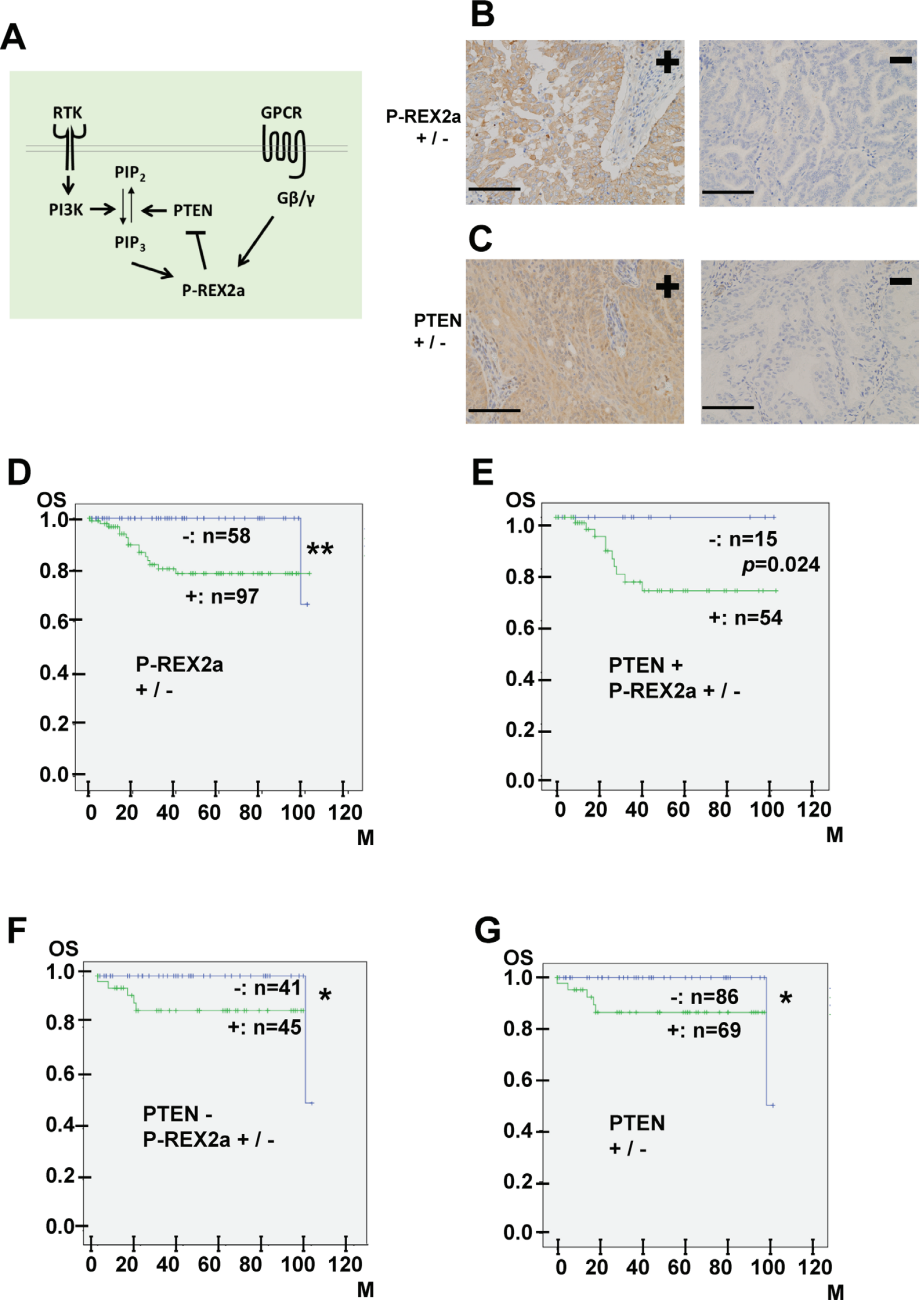
(**A**) A scheme showing the molecular interaction of P-REX2a. RTK, receptor tyrosine kinase, PI3K, phosphoinositide 3-kinase; PIP2, phosphatidylinositol-4,5-bisphosphate; PIP3, phosphatidylinositol-3,4,5-trisphosphate; PTEN; phosphatase and tensin homolog; P-REX2a; PIP3-dependent Rac Exchanger 2a; GPCR, G protein coupled receptor; Gβ/γ, beta-gamma subunits of heterotrimeric G protein. (**B**, **C**) Immunohistochemistry of 155 endometrial malignancies. Representative results for either positive (left) or negative (right) immunostaining for P-REX2a (B) or PTEN (C) are shown. See Materials & Methods for the criteria of scoring of the immunostaining results. Positive P-REX2a immunostaining (B, left). Cytoplasmic staining in more than 10% of the tumour cells is observed. Negative P-REX2a staining (B, right). Positive PTEN immunostaining (C, left). Negative PTEN staining (C, right). Scale bar (black line); 100 μm. (**D**–**G**) Kaplan-Meier analysis. P-REX2a expression is associated with worse overall survival in patients with endometrial malignancies. Kaplan-Meier survival curves showing overall survival in patients with endometrial malignancies. (D) P-REX2a-positive (green line) or P-REX2a-negative (blue line). (E) P-REX2a positive (green line) or PREX2a-negative (blue line) in the PTEN -positive cases. (F) P-REX2a-positive (green line) or P-REX2a-negative (blue line) in the PTEN-negative cases. (G) PTEN-positive (green line) or PTEN-negative (blue line) among the 155 cases. ^*^P < 0.05, ^**^P < 0.01, OS: overall survival rate, M: months.

Phosphatidylinositol-3,4,5-trisphosphate RAC exchanger 2a (P-REX2a) is a member of the P-REX protein family which are activated by phosphatidylinositol-3,4,5-trisphosphate that act as Rho/Rac guanine nucleotide exchange factors [7]. P-REX2 was at first found to activate the small GTPase Rac, downstream of G protein-coupled receptors and phosphoinositide 3-kinase (PI3K) to enhance cell motility [8]. Later, a new role of P-REX2a had been identified to inhibit the lipid phosphatase activity of PTEN to stimulate the PI3K pathway and enhance cell viability, movement and growth [9]. Among the P-REX family, which has P-REX1, P-REX2a, and P-REX2b as members, P-REX1 is mainly expressed in peripheral blood leukocytes or the brain, and P-REX2 is found in various human tissues such as skeletal muscle, small intestine, heart, lung, and placenta [7]. P-REX2a and P-REX2b are isoforms of PREX2. The size of the P-REX2a protein is 182 kDa, larger than that of P-REX2b, 111 kDa, because P-REX2b lacks the C-terminal domain of P-REX2a [7]. Known molecular interactions of P-REX2a are briefly summarized in Figure 1A.

Recently, mutations in the P-REX2a gene has been reported in human malignant melanoma and pancreatic cancer, suggesting a tumorigenic role for P-REX2a [10, 11]. and while P-REX1 expression in breast cancer has been associated with worse prognosis of patients, genomic changes in P-REX1 or P-REX2 have been found in various human carcinomas other than melanoma such as in breast, prostate, colorectal, and lung cancer [7]. However, no association with P-REX2a and human endometrial cancer has been reported to date. Furthermore, P-REX2a may contribute in endometrial tumor progression independent with PTEN inhibition. Thus, in this study, we immunohistochemically analysed a total of 155 cases of human endometrial neoplasms for P-REX2a expression and focused on its relationship with patient prognosis together with the underlying molecular mechanisms.

## RESULTS

### Immunohistochemical expression of P-REX2a in endometrial tumors

First, we examined the expression of P-REX2a in 155 primary human endometrial tumour tissues by immunohistochemistry. Figure 1B shows images of the positive and negative staining results. Cytoplasmic staining in >10% of the tumour cells was considered positive, and the others, negative, which was readily reproduced. Of the 155 cases, 132 were endometrioid carcinomas, 14 carcinosarcomas, and 2 each of serous carcinomas, low-grade endometrial stromal sarcomas, and squamous cell carcinomas. The other cases included 1 each of undifferentiated carcinoma, neuroendocrine carcinoma, and mixed carcinoma. In total, 97 (63%) cases were P-REX2a-positive and 58 cases (37%) were P-REX2a-negative. Next, we examined PTEN expression. Of the 155 cases, 69 (45%) were positive for PTEN and 86 (55%) were negative. Positive cytoplasmic PTEN immunostaining was observed in 69 of the 155 cases (Figure 1C, left), and 86 cases were negative (Figure 1C, right).

### P-REX2a-positive endometrial malignancies have worse prognoses, independent of PTEN expression

To further evaluate the role of P-REX2a, we analysed the relationship between P-REX2a expression and the OS of patients using the Kaplan-Meier method together with the log-rank test. The P-REX2a-positive group had significantly shorter OS (*P* = 0.003, Figure 1D) rate than that of the P-REX2a-negative group. Then, we subdivided the cases into 2 groups, PTEN-positive or PTEN-negative, and analysed the survival of patients. The Kaplan-Meier curves for PTEN-positive (Figure 1E) or PTEN-negative (Figure 1F) cases demonstrated worse prognosis in the P-REX2a-positive patients, and among them, the PTEN-negative group (Figure 1F) exhibited significant differences (*p* = 0.024). Regardless of P-REX2a expression, the PTEN-negative cases tended to have significant better prognoses (Figure 1G) (*p* = 0.012). Then, we analysed factors that contributed to prognosis by using univariate and multivariate Cox proportional hazards models for further validation. Statistical assessment of both univariate and multivariate analyses showed that P-REX2a expression and grade of the endometrioid carcinoma cases were significantly associated with OS but not PTEN expression or FIGO stage (Table 1). These results indicated that P-REX2a expression promoted progression of endometrial malignancies independently of PTEN expression.

**Table 1:** Univariate and multivariate analysis of risk factors

Univariate analysis				
	Hazard Ratio	Lower 95% Confidence Limit	Upper 95% Confidence Limit	*p*-value
Grades of endometrioid carcinoma	6.312	2.152	18.51	0.000792^***^
P-REX2a positive immunostaining	11.78	1.52	91.27	0.01824^*^
PTEN positive immunostaining	2.364	0.8543	654	0.0976
FIGO Stage	1.331	0.982	1.804	0.06532

Multivariate analysis				
	Hazard Ratio	Lower 95% Confidence Limit	Upper 95% Confidence Limit	*p*-value
Grades of endometrioid carcinoma	5.747	1.877	17.59	0.002193^**^
P-REX2a positive immunostaining	9.573	1.164	78.77	0.03566^*^
PTEN positive immunostaining	1.427	0.4677	4.353	0.5323
FIGO Stage	1.073	0.7439	1.546	0.7076

^***^p < 0.001, ^**^p < 0.01, ^*^p < 0.05.

### P-REX2a knockdown decreases endometrioid carcinoma cell mobility

**Figure 2:**
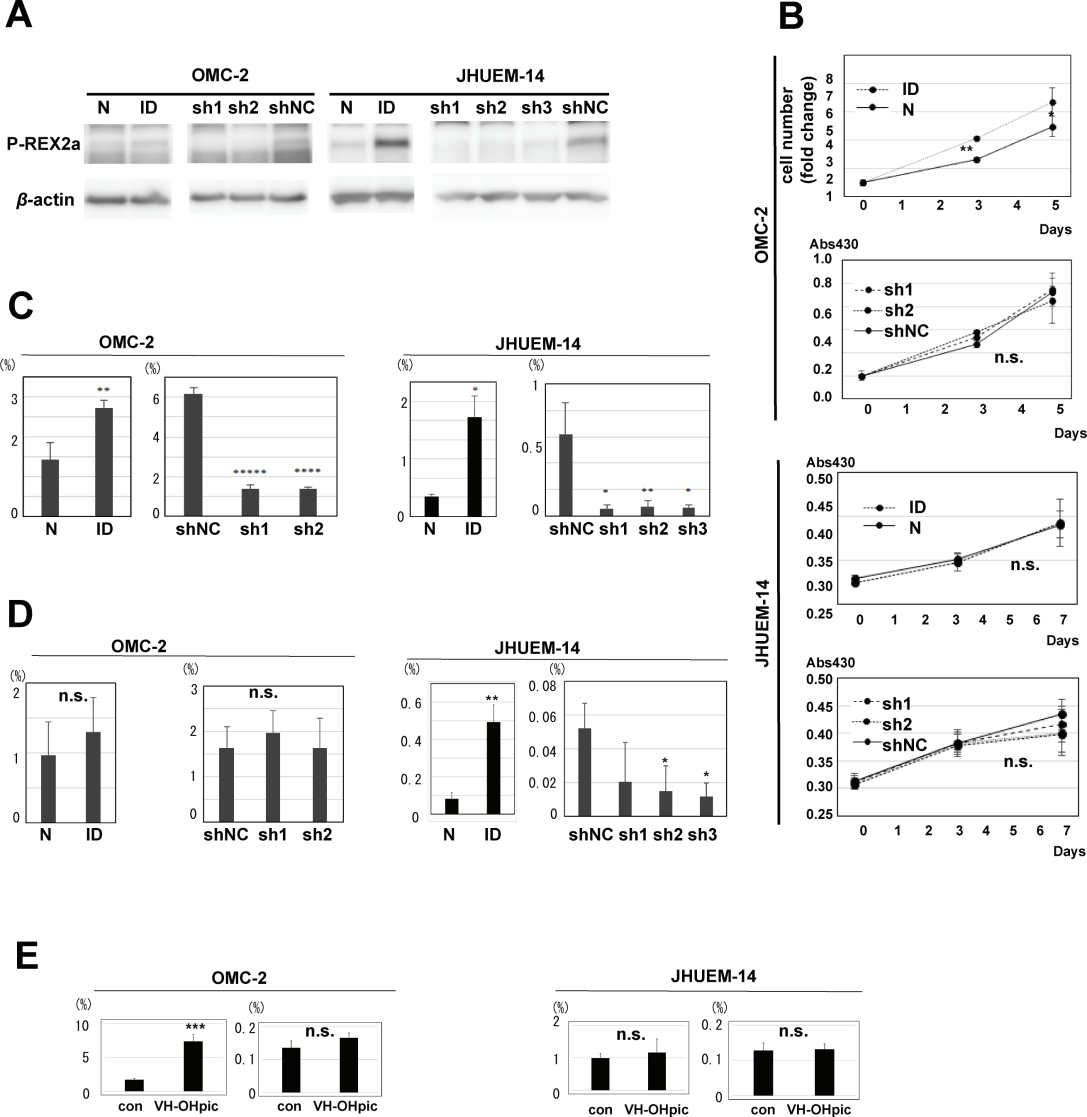
Role of P-REX2a in endometrial carcinoma cell lines (**A**) Immunoblotting for P-REX2a in OMC-2 and JHUEM-14 cell lines. P-REX2a-induced (ID) or control (N) cells, and knockdown of P-REX2a by 3 short hairpin RNAs targeting P-REX2a (sh1, sh2, sh3) or control (shNC). (**B**) Changes in cell number and cell viability of P-REX2a-expressing (ID) OMC cells or P-REX2a-silenced (sh1, sh2) OMC or JHUEM-14 cells and control (N, shNC). ^*^P < 0.05, ^**^P < 0.01. n.s., not significant; *p* > 0.05. (**C**, **D**). The results of cell migration (C) and invasion (D) assays using the cells mentioned above. ^*^P < 0.05, ^**^P < 0.01, ^***^P < 0.001, ^****^P < 0.0001, ^*****^P < 0.00001. n.s., not significant; *p* ≥ 0.05. (**E**) Effects of a PTEN inhibitor on cell migration of OMC-2 and JHUMEC-14. con, control, VH-OHpic, a PTEN inhibitor, ^****^P < 0.0001, n.s., not significant; *p* ≥ 0.05.

Because immunohistochemical analysis demonstrated that P-REX2a-positive human endometrioid carcinomas were more aggressive, we performed *in vitro* experiments to validate the results. We used 2 different endometrioid carcinoma cell lines, OMC-2 and JHUEM-14. Both cell lines express P-REX2a; however, the OMC-2 cells have low P-REX2a expression compared with that of JHUEM-14 cells as detected by Western blotting (Figure 2A). Then, retroviral transduction of p-REX2a was performed for OMC-2 cells, and successful expression of the P-REX2a protein was observed in this cell line (Figure 2A). We also infected the 2 cell lines with retroviruses expressing 2 shRNAs targeting P-REX2a, together with control, to perform knockdown experiments *in vitro*. Efficient knockdown of P-REX2a by shRNAs in both cells was confirmed by Western blotting (Figure 2A). Then, we compared P-REX2a-expressing OMC-2 cells and control cells for changes in cell number and viability. Although P-REX2a expression in OMC-2 cells resulted in significant increase in both cell number and viability, the decrease in cell number was not significant after P-REX2a knockdown in either of the 2 cell lines (Figure 2B). Then, we performed cell migration and invasion assays using all these cell lines along with appropriate controls. P-REX2a induction significantly increased cell mobility in both cell lines, and P-REX2a knockdown significantly decreased cell migration in all cell lines (Figure 2C). P-REX2a induction significantly increased and P-REX2a knockdown decreased cell invasion in JHUEM-14 cells, however, P-REX2a induction nor knockdown did not affect cell invasion in OMC-2 cells (Figure 2D). Finally, we applied a PTEN inhibitor to seek for the role of PTEN in cell viability or migration of these cells. The motility of OMC-2 cells was significantly enhanced and the viability also slightly increased (not significantly) by PTEN inhibition. In contrast, no difference was observed in either assay in JHUEM-14 cells, confirming PTEN dysfunction due to lack of expression in the cell line (Figure 2E).

### P-REX2a controls cell migration via GPCR downstream pathway

**Figure 3:**
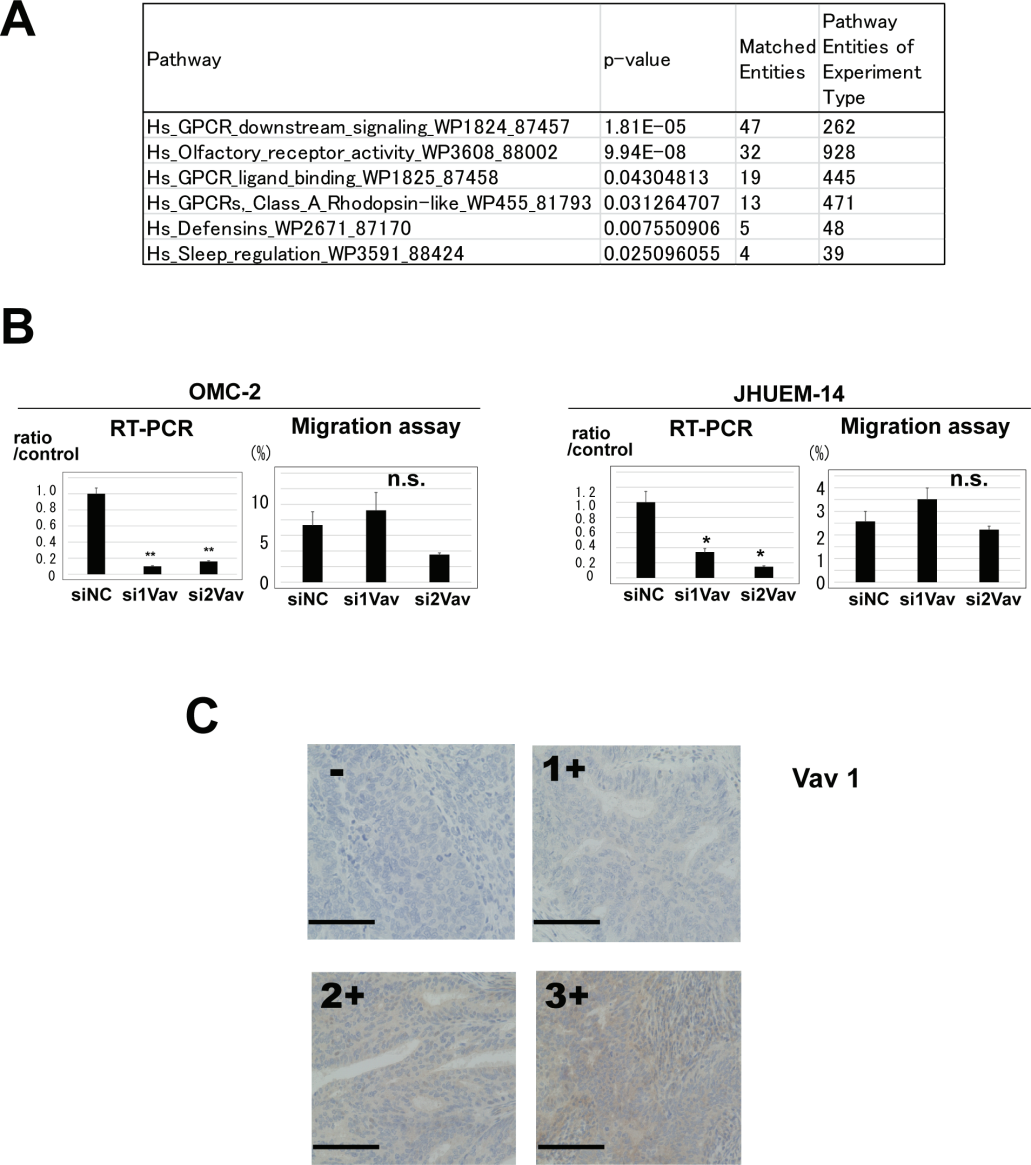
(**A**) Pathway analysis of 1,882 genes that displayed elevated expression levels by more than 2-fold and *p*-values less than 0.05 compared to the P-REX2a-knockdown groups and control using GeneSpring GX 13.1 software. Pathways with matched entities with *p*-values less than 0.05 are shown. (**B**) Vav1 knockdown experiments using OMC-2 and JHUEM-14 cells. Quantitative reverse-transcription PCR shows effective knockdown of Vav1 in both cell lines with 2 different siRNA sequences (left), but no significant changes are observed in migration assays (right). RT-PCR, reverse-transcription PCR; ^**^P < 0.01, ^*^P < 0.05, n.s, not significant; *p* ≥ 0.05. (**C**) Immunohistochemistry for Vav 1. A case of negative immunostaining is shown on the upper left. Positive cytoplasmic immunostaining was further quantified and scored into 3 levels, 1+ to 3+. See Materials & Methods for the criteria of the immunostaining. Scale bar (black line); 100 μm.

Then, we performed expression microarray analysis to identify the molecular mechanism by which P-REX2a contributes in increased cell mobility. The microarray data are publicly available (GSE98560). First, by comparing the genes that exhibited more than 4-fold change together with less than 0.05 *p*-value due to P-REX2a expression, we identified 377 genes (Supplementary Table 1), and subsequent pathway analysis revealed that these genes were involved in various pathways associated with human cancers (Supplementary Table 2). In addition, we identified 1,882 genes for which the expression levels were significantly different in the P-REX2a-knockdown groups in both cell lines by more than 2-fold compared to the control groups and the statistical values were *P* < 0.05 (Supplementary Table 3). We performed pathway analyses in these genes and found that only 6 pathways were significantly (*p* < 0.05) involved, and among them, 4 were associated with the GPCR signalling pathway including the olfactory receptor activity group, which is also a member of the GPCR superfamily (Figure 3A). Olfactory receptors, OR6N1 and OR2D2, were significantly down-regulated by the knockdown of P-REX2a. Within the 4 pathways, the GPCR downstream signalling pathway had the most matched genes. Vav1, a member of the GPCR downstream signalling pathway, which is known to have a positive role together with P-REX1 in up-regulating cell migration [12, 13], was detected by microarray analysis in both of cell lines and down-regulated by P-REX2a knockdown, albeit the change was not significant. By quantitative RT-PCR, Vav1 mRNA expression was detected in both cell lines, OMC-2 and JHUEM-14, which were significantly reduced by siRNA induction (Figure 3B). However, knockdown of Vav1 had no significant effect in reducing cell mobility in either of the cells (Figure 3B). Then, we analyzed Vav1 expression using a tissue microarray containing human endometrial tumor samples (Figure 3C). Interestingly, Vav1 expression displayed a weak but significant positive correlation with the expression of P-REX2a (*r* = 0.44, *p* < 0.001), which suggests that, in association with Vav1, P-REX2a contributes towards worse prognosis in patients via the GPCR pathway to enhance cell mobility, resulting in earlier metastasis of tumor cells to the ovaries or into the peritoneal cavity.

## DISCUSSION

In this study, we demonstrated for the first time that P-REX2a expression is related to the worse behaviour of endometrial malignancies. Approximately 85% of our cases were endometrioid carcinomas. The endometrioid carcinomas are subdivided 2 Bokhman subtypes, type I and type II [1]. Negative PTEN expression has been previously reported to be associated with better prognosis of endometrial carcinoma patients, related to PTEN mutation, which occurs more frequently in type I endometrial cancer [1, 6]. In our analysis, PTEN expression was also significantly associated with worse prognosis, but was not an independent prognostic factor. However, P-REX2a expression was independently associated with worse prognosis of patients. Furthermore, increase in cell motility was seen in cells lacking PTEN expression, thus confirming the *in vivo* data.

*In vitro* data from this study indicated that P-REX2a is more important in cell migration than invasion. The reason for this discrepancy remains unclear. A disintegrin and metalloproteinase (ADAM) 19 was significantly down-regulated by P-REX2a knockdown, and since ADAMs are known to be involved in cancer cell proliferation and progression [14], ADAMs may have some role in cell motility in this type of tumor. However, no significant change was observed in the expression of matrix metalloproteinases (MMPs), which are important in the destruction of basement membranes [15]. Therefore, these endometrial cancer cells may have had some limitation in invading the Matrigel used in our experiment. Biologically, the human endometrium-myometrium interface lacks an intervening tissue layer such as a fibre-rich basement membrane, and carcinoma cells or normal endometrial epithelial cells together with stromal cells can easily invade the myometrium [16]. Thus, endometrial tumours may invade the myometrium, migrate within the uterine cervical canal or fallopian tubes to invade the endocervix or metastasize to the ovaries and may even disseminate into the peritoneal cavity without tissue destruction. Therefore, it is important to consider the cell type specificity for better understanding the biological differences between endometrial cancers and carcinomas of other organs such as the breast or pancreas.

GPCRs constitute the largest group of cell surface receptors in the human body [17], and the olfactory receptor family is also a member of the GPCR superfamily [18]. In this study, 2 olfactory receptors, OR6N1 and OR2D2, showed significant changes in mRNA expression in response to P-REX2a knockdown, and other than the mRNAs in the olfactory receptor group, the remaining mRNAs were mostly members of the GPCR downstream signalling pathway or GPCR ligand binding groups. Although we demonstrated that the expression of Vav1, a member of the GPCR downstream signalling pathway that was previously reported to cooperate with P-REX1 in murine neutrophils [12, 13] correlated with P-REX2a expression in the clinical samples. Vav proteins are recently known as a family of 3 proteins, Vav1, Vav2, and Vav3, having functional redundancy at least in some occasions [19]. In our experiment, Vav1 knockdown had no significant effects in cell migration. Because all Vav1, Vav2, and Vav3 were detected by RT-PCR in our endometrial carcinoma cell lines (data not shown), functional compensation may have occurred. However, we could not clarify the direct mechanism, and our data could not show direct evidence that Vav1 functionally cooperates with P-REX2a in endometrial neoplasms. We also could not specify the type of the GPCR or all of the molecules involved downstream. Further studies are needed for revealing each step leading to the enhanced cell motility and aggressive behavior of endometrial tumors, which would be useful for drug development.

## MATERIALS AND METHODS

### Patients and tissue samples

Formalin-fixed, paraffin-embedded tumor samples from 155 primary endometrial malignancies were obtained from patients who underwent surgical treatment at Nagoya City University Hospital from January 2004 to December 2012 and had clinical follow-up information available. All histologic diagnoses were reviewed by 3 experts in diagnostic pathology, YY, AN, and SaTa. Tumour staging was based on the International Federation of Gynaecology and Obstetrics (FIGO) classification [20]. All patients were primarily treated with optimally debulking surgery by skilled surgeons in gynecologic oncology. Two (1.3%) of the 155 patients received neoadjuvant chemotherapy, and one was treated with pre-operative radiation. All samples were obtained from Nagoya City University Hospital with informed consent. The experimental design of genomic and expression studies was reviewed and approved by the Committee for Bioethics of Nagoya City University.

### Immunohistochemistry

Formalin-fixed, paraffin-embedded blocks were sectioned into 3–5 µm slices for immunohistochemistry similarly as previously described [21]. Tissue microarray blocks were generated by removing a 2-mm core from each tumor. Anti-P-REX2a (ab121462, Abcam, Cambridge, MA, USA), anti-PTEN (ab21873, Abcam, Cambridge, UK), and anti-Vav1 (#PA5-21495, Thermo Fisher Scientific, Waltham, MA, USA) primary antibodies were used. Immunostaining was interpreted by three independent pathologists blinded to the clinical data. For P-REX2a, first, the samples that displayed cytoplasmic staining in more than 20% or 10% of cancer cells were determined as 2- or 1-plus, respectively. The 2-plus and 1-plus samples were considered positive, and the others, negative. Positive immunostaining of PTEN was observed in the cytoplasm or nuclei of tumour cells, and although the results of preliminary analyses were similar because PTEN localizes near the cell membrane, we considered cytoplasmic immunostaining to be positive. For Vav1, positive immunostaining was seen only in the cytoplasm of tumour cells, and the signal intensities were classified into 3 categories. For correlation analysis, positivity scores were applied, which included 2 categories for P-REX2a and 3 for Vav1.

### Cell culture and cell lines

Two endometrioid adenocarcinoma cell lines established from human endometrial tumors, JHUEM-14 and OMC-2 were used in this study. The JHUEM-14 cell line was obtained from the Riken Bioresource Center (BRC, Tsukuba, Ibaraki, Japan), and the OMC-2 cell line was a generous gift from Dr. Takashi Yamada (Osaka Medical University, Takatsuki, Japan) [22]. The cells were cultured in RPMI-1640 medium (Sigma-Aldrich, St Louis, MO, USA) containing 10% foetal bovine serum at 37° C in a 5% CO2 atmosphere. Both cell lines were tested and authenticated using the short tandem repeat (STR) method [23]. For PTEN inhibition, a PTEN inhibitor; VO-OHpic trihydrate (ab141439, Abcam, Cambridge, UK) was used with a pre-evaluated concentration at 1 μM.

### Construction of plasmids and viral transduction for expression and knockdown of P-REX2a

Ectopic expression and knockdown of P-REX2a were performed as previously described [24]. Briefly, construction of the human P-REX2a-encoding retroviral vector was performed using the Gateway system to transfer the coding region of P-REX2a from commercially available plasmid DNA (human Prex2 transcription variant 1 (NM_024870.2) #GC-Z4890, GeneCopoeia, Rockville, MD, USA) to a destination vector, and retroviruses were produced by transient transfection. To knock down human P-REX2a, short hairpin RNA (shRNA)-encoding retroviral vectors were transfected into cells using retroviral vectors encoding the human H1 promotor. The sense coding sequences of the shRNAs used are as follows: shPrex2a-1, GCTAATGTGTGGAGTCTTA; shPrex2a-2, GGAGATGTGTGTTTGTCAA; shPrex2a-3, GCTCCTGAATGCTGGACTA; and shNC, ATCTGAAGACCTATTTTAT.

### Vav1 knockdown and reverse-transcription (RT)-PCR

To knockdown Vav1, 2 different sequences of short-interffering RNA (siRNA) and a negative control were used, namely, Silencer^®^ Select Pre-designed siRNA (si1Vav s14750, si2Vav: s14751, Ambion^®^ Thermo Fisher Scientific) and Mission siRNA Universal Negative Control (Sigma-Aldrich, St.Louis, MO, USA). Cells were transfected using Lipofectamine™ RNAiMAX Transfection Reagent (Invitrogen^®^ Thermo Fisher Scientific). RNA extraction, cDNA synthesis and quantitative reverse transcription (RT)-PCR was performed as previously described [25].

### Western blot analysis

Immunoblotting was performed using whole-cell lysates as previously described [24]. Samples containing 20 µg proteins were separated using sodium dodecyl sulphate polyacrylamide gel electrophoresis and blotted on Immobilon P filters (Millipore, Billerica, MA, USA). The following antibodies were used: anti-Prex2 (ab121462, Abcam), anti-PTEN (#9188, Cell Signaling Technology, Beverly, MA, USA), and anti-β-actin (1:2000) (A5316, Sigma-Aldrich). We confirmed that the cell lines and human endometrial tumors samples expressed P-REX2a and not P-REX2b by examining the sizes of the bands in the Western blots.

### Cell proliferation and viability assay

Cells were cultured for 5 or 7 days, and the cell numbers were determined by a Coulter counter at 0, 3, and 5 or 7 days. For viability assays, cells were incubated in 96-well plates in 5% CO_2_ at 37° C. After 24, 48, 72 and 96 h, cell viability was assayed by WST assay using the CellTiter 96 Aqueous One Solution Cell Proliferation Assay kit (Promega, Madison, WI, USA) according to the manufacturer’s instructions.

### Migration and invasion assays

The assays were performed using 6.5 mm Transwell plates with 8.0 µm pore polycarbonate membrane inserts (Corning Coaster, Rochester, NY, USA). For the invasion assay, the upper surfaces of the filters were coated with 50 µl of Matrigel (Becton and Dickenson, Franklin Lakes, NJ, USA). Next, 1 × 10^5^ cells were seeded in the upper chambers in culture medium without FBS with 10% FBS in the lower chambers. The cells were incubated for 24 h at 37° C in 5% CO_2_. After removing the non-invaded or non-migrated cells, the remaining cells were stained with Giemsa.

### Expression microarray analysis

Expression microarray analysis was performed similarly as previously described [25] using OMC-2 and JHUEM-14 cells with P-REX2a knockdown or control and OMC-2 cells with P-REX2a expression or control. Experiments were performed in duplicate; thus, total RNA extracted from 12 samples was reverse transcribed and labelled using a Low Input Quick Amp Labelling Kit, One-colour (Agilent Technologies, Santa Clara, CA, USA) and hybridized to a Sureprint G3 Human Microarray, 8 × 60 K Ver 3.0, G4851C; Agilent Technologies). The analysis was performed using GeneSpring GX 13.1 software (Agilent Technologies).

### Statistical analysis

Chi-squared tests were used to evaluate the associations between P-REX2a expression status and clinicopathological factors. Overall survival (OS) was calculated from the date of surgery to the date of last follow-up or date of death from endometrial malignancies. Survival analyses were performed using the Kaplan-Meier method to estimate OS, and statistical significance was determined using the log-rank test. A multivariate analysis was performed using a Mantel-Cox proportional hazards model. Student’s *t*-test (for comparison of the 2 groups), one-way ANOVA or two-way ANOVA (for multiple comparisons) was used to evaluate the numerical data. For analysis of the microarray data, Student’s *t*-test was used.

## SUPPLEMENTARY MATERIALS TABLES







## Abbreviations

P-REX2a: PIP3-dependent Rac Exchanger 2a
PTEN: phosphatase and tensin homolog
Rac: ras-related C3 botulinum toxin substrate
GPCR: G protein coupled receptor
Vav 1: vav guanine nucleotide exchange factor 1
PI3K: phosphoinositide 3-kinase
FIGO: International Federation of Gynaecology and Obstetrics
STR: short tandem repeat
shRNA: short-hairpin RNA
siRNA: short-interfering RNA
OS: overall survival
ADAM: A disintegrin and metalloproteinase
MMP: matrix metalloproteinase
