# Supplementary material for: Expression of P-REX2a is associated with poor prognosis in endometrial malignancies
Source: Oncotarget. 2018 May 15;9(37):24778–86. doi: 10.18632/oncotarget.25349 (PMC5973852; doi:10.18632/oncotarget.25349)
Supplement: Supplementary file 1 [file oncotarget-09-24778-s001.pdf]

Expression of P-REX2a is associated with poor prognosis in endometrial malignancies

SUPPLEMENTARY MATERIALS

Supplementary Table 1: List of the 377 genes with significant expression changes by P-REX2a induction ($p < 0.05$, fold change > 4.0). See Supplementary_Table_1

Supplementary Table 2: Pathway analysis of the 377 genes

Pathway	*p*-value	Matched Entities	Pathway Entities of Experiment Type	Organism
Hs_Integrated_Pancreatic_Cancer_Pathway_WP2377_84983	5.57E-04	7	201	Homo sapiens
Hs_MAPK_Signaling_Pathway_WP382_79951	0.006901779	5	168	Homo sapiens
Hs_AGE-RAGE_pathway_WP2324_78487	1.05E-04	5	66	Homo sapiens
Hs_EGF-EGFR_Signaling_Pathway_WP437_79266	0.001054903	6	162	Homo sapiens
Hs_Retinoblastoma_(RB)_in_Cancer_WP2446_87639	2.88E-06	7	90	Homo sapiens
Hs_Nucleotide_Metabolism_WP404_68960	0.007786888	2	19	Homo sapiens
Hs_Eicosanoid_Synthesis_WP167_89514	0.008612175	2	25	Homo sapiens
Hs_Cell_Cycle_WP179_89516	0.006149875	4	103	Homo sapiens
Hs_Osteoblast_Signaling_WP322_79496	0.004241015	2	14	Homo sapiens
Hs_Osteoclast_Signaling_WP12_88602	0.00554092	2	16	Homo sapiens
Hs_Fluoropyrimidine_Activity_WP1601_84700	0.001468732	3	34	Homo sapiens
Hs_ABC-family_proteins_mediated_transport_WP1780_86383	0.003928342	3	45	Homo sapiens
Hs_TP53_Regulates_Transcription_of_Cell_Cycle_Genes_WP3804_88397	9.14E-04	4	62	Homo sapiens
Hs_Arachidonic_acid_metabolism_WP2650_87085	0.005287802	3	53	Homo sapiens
Hs_Mitotic_Prometaphase_WP2652_87985	0.006361126	4	110	Homo sapiens
Hs_Apoptotic_execution_phase_WP1784_86436	0.005287802	3	52	Homo sapiens
Hs_Mitotic_G1-G1-S_phases_WP1858_86357	5.31E-06	8	136	Homo sapiens
Hs_DNA_Damage-Telomere_Stress_Induced_Senescence_WP3565_87144	0.00917519	3	61	Homo sapiens
Hs_Regulation_of_Hypoxia-inducible_Factor_(HIF)_by_oxygen_WP2727_88138	3.71E-05	4	29	Homo sapiens
Hs_Nuclear_Receptors_Meta-Pathway_WP2882_87959	0.006031284	7	318	Homo sapiens
Hs_Mammary_gland_development_pathway_-_Involution_(Stage_4_of_4)_WP2815_88702	0.002136538	2	10	Homo sapiens

Supplementary Table 3: List of the 1,882 genes with significant expression changes by P-REX2a knockdown ($p < 0.05$, fold change > 2.0). See Supplementary_Table_3
